# Scoping Review: Is Push-Dose Norepinephrine a Better Choice?

**DOI:** 10.5811/westjem.18584

**Published:** 2024-07-17

**Authors:** Michael Berkenbush, Lali Singh, Kelly Sessa, Raghad Saadi

**Affiliations:** Morristown Medical Center, Sameth Emergency Department, Morristown, New Jersey

**Keywords:** push-dose vasopressors, norepinephrine, anesthesia-induced hypotension, critical care

## Abstract

**Introduction:**

The use of push-dose vasopressors to treat anesthesia-induced hypotension is a common evidence-based practice among anesthesiologists. In more recent years, the use of push-dose vasopressors has transitioned to the emergency department (ED) and critical care setting. There is debate on the best choice of a push-dose vasopressor, with push-dose epinephrine or phenylephrine being more commonly used. This scoping review evaluated publications regarding the clinical use of push-dose norepinephrine.

**Methods:**

We queried research studies in both PubMed and Google Scholar on the use of push-dose norepinephrine in human subjects, with numerous randomized controlled trials that compare norepinephrine to other vasopressors including phenylephrine, ephedrine, and epinephrine.

**Results:**

A large majority of the studies were performed in the setting of spinal anesthesia prior to cesarean section, while several involved the administration of general anesthesia, with limited-to-no literature in the emergency and critical care setting. Of the 27 studies that we included in the review, 17 were randomized controlled trials. These studies demonstrated that norepinephrine was safe and effective.

**Conclusion:**

Prior research has demonstrated the superiority of norepinephrine as a pressor of choice for various shock states. In this review, the safety and efficacy of push-dose norepinephrine is demonstrated, and favorable hemodynamic markers are shown in comparison to other agents. In addition, there are some safety and efficiency benefits to using push-dose norepinephrine from an administration standpoint, as well as clinically in decreased need for repeat doses. Further high-quality studies in the emergency and critical care realm would be beneficial to confirm these findings.

Population Health Research Capsule What do we already know about this issue?

*Push-dose vasopressors are commonly used in emergency and critical care settings. Norepinephrine infusions have been shown to be superior to other vasopressors.*

What was the research question?

*We sought to determine the evidence for push-dose norepinephrine compared to other vasopressors.*

What was the major finding of the study?

*The safety and efficacy of push-dose norepinephrine at 4–16 mcg is demonstrated compared to other agents.*

How does this improve population health?

*Push-dose vasopressors are a crucial part of emergency and critical care and are used for hemodynamic stabilization during time-sensitive patient emergencies.*

## INTRODUCTION

The use of push-dose vasopressors to treat anesthesia induced-hypotension is a common, evidence-based practice among anesthesiologists,^1^ which has transitioned to the emergency department (ED) and critical care setting. Push-dose vasopressors allow clinicians to urgently stabilize patients’ hemodynamics and provide additional time for procedures (eg, intubations) and bridging to continuous infusions. Norepinephrine (NE) is the vasopressor of choice in most shock states due in part to a decreased incidence of side effects,^2^ superior hemodynamic profile,^3^ and better control of the shock state.^4^ Our main purpose in this scoping review was to determine the evidence for push-dose NE compared to other vasopressors while addressing the safety, effectiveness, and efficiency of the drug.

## METHODS

In this study we evaluated full-text publications in the English language regarding the clinical use of push-dose NE in human subjects in accordance with PRISA-ScR guidelines. Using PubMed and Google Scholar in October 2022, literature was reviewed based on the following keywords: “bolus norepinephrine,” “push-dose norepinephrine,” and various formulations of “norepinephrine/administration and dosage” [Mesh] AND (push bolus OR bolus dose) Filters: English, Humans. Using OR including various other names for norepinephrine including Droxidop, Nordefrin, Normetanephrine, Levonorepinephrine, Noradrenaline, Levarterenol, Levonor, Levophed, Levophed Bitartrate, or Noradrénaline tartrate renaudin.

We identified 88 articles using these search terms (Figure). These articles were reviewed to determine whether the administration of NE was studied as a push or bolus dose. We excluded those which only included NE infusions. One reviewer reviewed the studies for eligibility, which was then confirmed independently by a second reviewer. Non-human studies, case reports, and letters to the editors were excluded. We also excluded studies on circulating plasma levels of NE, as well as articles whose primary outcome was not hemodynamics. One excluded article focused on NE use to avoid post-reperfusion syndrome in liver transplant, and another focused on umbilical arterial pH in neonates after their mothers received NE during their cesarean section. Of these included articles, 27 studies that included push-dose NE were included in the following review. The articles obtained were published in or after 2015 except for one study.^5^ Seventeen were randomized controlled trials (RCT), and four were dose-finding trials. There were three prospective observational studies and three literature reviews.

**Figure.**
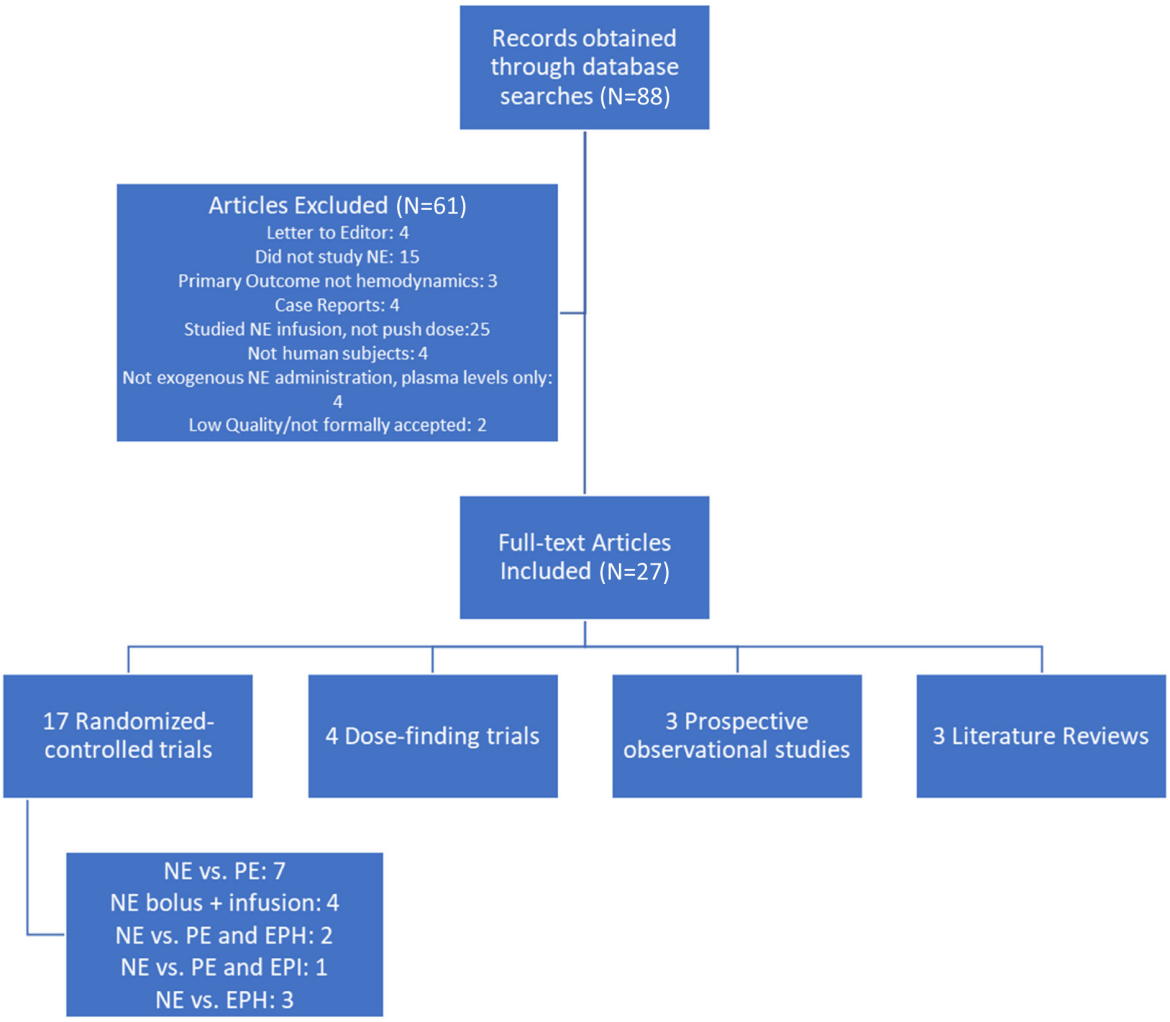
Search Strategy and Results. *NE*, norepinephrine; *PE*, phenylephrine; *EPH*, ephedrine; *EPI*, epinephrine.

## RESULTS

There were seven RCTs that compared NE with phenylephrine, encompassing a total of 626 patients, all in the setting of maternal patients receiving spinal anesthesia for cesarean section.^6^ ^–^ ^12^ Norepinephrine showed less risk of bradycardia compared to phenylephrine. The use of NE required fewer boluses than phenylephrine to correct hypotension and maintained higher cardiac output and stroke volume in comparison to phenylephrine. There were no safety concerns with NE used peripherally. The relative potency ratio used on average was norepinephrine:phenylephrine 1 microgram (μg):12.5 μg.

Two double-blinded RCTs compared NE with both phenylephrine and ephedrine.^13^ ^,^ ^14^ These two studies involved a total of 211 patients receiving spinal anesthesia for cesarean section. Norepinephrine had fewer episodes of bradycardia compared to phenylephrine and fewer episodes of tachycardia compared to ephedrine. In one study^14^ NE maintained a higher mean arterial pressure (MAP) compared to the other two agents.

Baraka et al looked at the hemodynamic effects in phenylephrine, NE, and epinephrine for intubated patients undergoing coronary artery bypass grafting^5^. Phenylephrine and NE had similar changes in systemic vascular resistance without significant change in cardiac output. All agents increased MAP similarly in this study. Of note, this was the only study we found that compared push-dose epinephrine with NE.

Three double-blinded RCTs compared NE with ephedrine with 276 total patients.^15^ ^–^ ^17^ In each study, NE was found to be more efficacious than ephedrine in maintaining MAP and was also associated with less tachycardia and fewer total doses given. One study used radial artery catheters for monitoring both MAP and heart rate, while the others used non-invasive monitoring at standard intervals. These studies also included hypertensive patients undergoing spinal surgery and patients with coronary artery disease undergoing knee arthroscopy.

Four RCTs compared different NE boluses/infusions, two of which were double-blinded.^18^ ^–^ ^21^ Each of these studies was related to spinal-induced hypotension during cesarean section, and primarily focused on prevention of hypotension by administration of prophylactic NE. Infusions were successful in preventing hypotension, and higher boluses (6 μg or 0.10 μg/kg) performed better compared to lower boluses (4 mcg or 0.05 μg/kg).

There were four dose-finding trials including a total of 342 patients undergoing spinal anesthesia during cesarean delivery.^22^ ^–^ ^25^ Different methods were used to determine efficacy, with the primary goal of maintaining systolic blood pressure at 80% of the initial reading. For a double-blind sequential allocation study with biased coin up and down design, the ED_90_ was 5.8 μg. Using random allocation graded dose response, the ED_50_ for NE was 10 μg. Another study comparing prophylactic versus rescue bolus of NE has an ED_90_ of 10.85 μg for prophylactic dose and 12.3 μg for rescue dose.

There were three prospective, observational studies regarding push-dose NE, one during general anesthesia and two during spinal anesthesia for cesarean sections.^26^ ^–^ ^28^ Two of these studies compared NE against phenylephrine.^27^ ^,^ ^29^ Norepinephrine resulted in increased stroke volume compared to phenylephrine. Other hemodynamic effects were similar between the two agents. Another study reviewed the use of NE compared to ephedrine, with NE showing higher MAPs and less tachycardia compared to ephedrine, also lower number of boluses needed for treatment of hypotension.^28^

There were three prior literature reviews identified.^29^ ^–^ ^31^ The first performed in 2018 identified nine full-text articles regarding the use of norepinephrine.^30^ At that time, it was determined that the efficacy of NE is similar to phenylephrine without adverse outcomes, shows improvement in cardiac output and decreased risk of bradycardia. A second review focused on the pharmacology of various vasopressors and their diverse clinical scenarios for use (intraoperative, periprocedural, bridge to vasopressor infusions, post-cardiac arrest, and anaphylaxis).^31^ This article also covered the safety and compounding concerns with the bedside mixing of vasopressors. Diluted concentrations have been shown to be safe peripherally, although central access is preferred. A third review focused on anesthesia use of vasopressors and addressing the pharmacology of common agents in detail.^32^

## DISCUSSION

Push-dose epinephrine or phenylephrine are commonly used vasopressors in the critical care setting for the treatment of hypotension.^32^ Epinephrine has both alpha and beta effects, thus acting as an inopressor. At lower doses (0.01 to 0.1 μg/kg/min), epinephrine stimulates beta-1 and beta-2 receptors and functions as an inotrope, and at higher doses (>0.1 μg/kg/min) it also stimulates alpha-1 causing vasoconstriction, thus increasing blood pressure. Phenylephrine is a pure alpha-1 agonist that increases arterial and venous tone, thus increasing blood pressure. Ephedrine has both alpha-1 and beta-1 receptor activity. It is a popular choice in the operating room due to its rapid onset (one minute) and longer duration of action (60 minutes).

Norepinephrine is an alpha-1 receptor agonist with moderate beta-1 activity and minimal beta-2 activity. It is an attractive agent because its hemodynamic effects are dominated by its alpha-1 activity, while its beta-1 activity provides just enough inotropy to maintain cardiac output. There are advantages to using push-dose NE compared to other vasopressors including safety, effectiveness, efficiency, and cost.

### Safety

The preparation of push-dose vasopressors is associated with a high risk for medication errors because these medications are usually mixed at the patient’s bedside for immediate use in stressful situations.^33^ ^,^ ^34^ Because of this high risk, it is recommended that these preparations be double checked by another healthcare professional prior to administration. Immediately after preparation, all syringes and bags that are used in the process should be labeled with the appropriate concentration and medication name to further prevent any medication errors.

Compared to compounding other push-dose vasopressors, an advantage to push-dose NE is that it can allow for limited preparation depending on premixed bag availability. If using a premixed bag 4 milligrams (mg)/250 milliliters (mL) to make push-dose NE, it would require no mixing, and theoretically less chance for compounding errors. For institutions that do not have premix NE 4 mg/250 mL, the preparation of push-dose NE can be mixed by withdrawing the 4 mL of NE from the 4mg/4 mL vial and injecting it into a 250 mL bag of normal saline. From the bag, withdraw 10 mL of NE-containing fluid into a 10 mL syringe. The final concentration would be 16 μg/mL of norepinephrine.

All the dose-finding studies reviewed are in pregnant women undergoing elective cesarean sections receiving spinal anesthesia. However, this data could be extrapolated to the critical care setting with the understanding that patients might require higher empiric doses. Based on several studies assessing push-dose NE, doses ranging from 4–16 μg were reported to be safe and effective in treating or preventing hypotension due to spinal anesthesia. An initial push-dose of NE 4–16 μg (0.25–1 mL) seems to be reasonable in the critical care setting.

### Effectiveness

Norepinephrine is the first-line vasopressor for almost all forms of shock, including undifferentiated shock, vasodilatory/septic shock, and cardiogenic shock. The 2021 Surviving Sepsis Campaign recommends NE as the first choice in patients with septic shock (Evans 2021).^35^ In the CENSER trial, patients who received early NE had better control of their shock state by the six-hour mark.^4^ Not only does NE improve blood pressure, but it also improves cardiac output.^36^ It was previously thought that NE might worsen organ hypoperfusion compared to other vasopressors. However, it has since been shown that in patients with severe septic shock, those who received NE had higher splanchnic circulation compared to those who received epinephrine.^37^

A study done by Martin et al showed that patients treated with NE had significantly lower hospital mortality compared to those treated with high-dose dopamine and/or epinephrine.^38^ For cardiogenic shock, one study showed that NE has a lower incidence of refractory shock compared to epinephrine.^39^ Norepinephrine has also been shown to have a decreased risk of causing arrhythmias compared to dopamine, which makes it preferred over other vasopressors, especially in a cardiogenic shock state.^40^ In the setting of hypovolemic or hemorrhage shock, there is a concern that vasopressors may compromise microcirculation and cause tissue ischemia due to excessive arteriolar vasoconstriction. However, a recent study by Harrois et al observed that NE preserved intestinal microcirculation during hemorrhagic shock in mice.^41^ Additionally, there was a study completed by Poloujadoff et al in rats, which showed that when using either a hypotensive or normotensive target for fluid resuscitation in hemorrhagic shock, rats that received NE infusions had improved survival compared to those who received fluids only.^42^

The use of push-dose NE can be extended to settings beyond that of shock states, such as prior to or during procedures that require hemodynamic optimization, such as endotracheal intubation or the induction of general anesthesia. Baraka et al compared the effects of NE, phenylephrine, and epinephrine on patients who were endotracheally intubated before undergoing elective coronary artery bypass grafting.^5^ They found that the NE group had the highest increase in MAP (42.9% vs 35% and 32.6%). In addition, the PrePARE trial has shown that a fluid bolus may not be enough to prevent cardiovascular collapse.^43^ Push-dose epinephrine has gained popularity in the emergency and critical care realm for this purpose. However, randomized trials comparing epinephrine to NE in this setting have not been performed.

### Efficiency and Cost

Using a single agent for both push-dose and continuous infusion requires overall less medications/materials than compounding two different agents, which in turn improves efficiency in critical care settings where every minute matters. Compared to phenylephrine 10 mg vials and epinephrine 1 mg vials, the average wholesale price of NE 4 mg vials is slightly more expensive for the required dose needed to prepare push-dose NE.^44^ However, in critical care settings push-dose vasopressors are commonly used as a bridge to an infusion; therefore, the NE cost would be negated, as the same bag used to make up the push-dose NE can be used for the infusion.

## LIMITATIONS

This review of push-dose NE demonstrated a significant number of double-blinded RCTs. However, nearly all the studies were performed in the operating theater, with limited data in the ED or critical care setting. Patients may be less optimized, and likely would be considerably older and have more comorbidities, than the populations of pregnant females primarily studied in this review undergoing spinal anesthesia. In addition, the optimal dosing of NE may not be accurately reflected for the critical care setting. However, the lack of adverse events is reassuring for its safety profile. It is unclear whether the shorter half-life of NE would impact its utility for periprocedural use; however, the effect of push-dose NE remained either equivalent or better than other vasopressors in the studies reviewed. Norepinephrine appears to be the vasopressor of choice compared to phenylephrine and ephedrine. Comparisons to push-dose epinephrine, which is more commonly used in the critical care setting, are also lacking.

## CONCLUSION

Prior research has demonstrated the superiority of norepinephrine as a pressor of choice for various shock states, although it is recognized that the clinical situation may dictate which vasopressor is used. In this review, the safety and efficacy of push-dose NE are demonstrated, and favorable hemodynamic markers are shown in comparison to other agents. In addition, there are some safety and efficiency benefits to using push-dose NE, from both an administration standpoint and clinically in a decreased need for repeat doses. Further high-quality studies in the emergency and critical care realm would be beneficial to confirm these findings.
